# Factors Associated With Adherence to Self-Managed Aphasia Therapy Practice on a Computer—A Mixed Methods Study Alongside a Randomized Controlled Trial

**DOI:** 10.3389/fneur.2020.582328

**Published:** 2020-11-23

**Authors:** Madeleine Harrison, Rebecca Palmer, Cindy Cooper

**Affiliations:** ^1^Division of Nursing and Midwifery, Health Sciences School, University of Sheffield, Sheffield, United Kingdom; ^2^Rehabilitation and Assistive Technology Group, School of Health and Related Research (ScHARR), University of Sheffield, Sheffield, United Kingdom; ^3^Clinical Trials Research Unit, School of Health and Related Research (ScHARR), University of Sheffield, Sheffield, United Kingdom

**Keywords:** aphasia, stroke, adherence, tele-rehabilitation, computer therapy, word finding

## Abstract

**Background:** Aphasia is a communication disorder often acquired after a stroke. Independent use of specialist aphasia software on a home computer is a form of asynchronous tele-rehabilitation that can provide increased opportunity for practice of rehabilitation exercises. This study aimed to explore the factors associated with adherence to self-managed aphasia computer therapy practice.

**Method:** A mixed methods exploration of adherence was conducted alongside the Big CACTUS randomized controlled trial [ISRCTN: 68798818]. The trial evaluated the clinical effectiveness of self-managed aphasia computer therapy. This study reports secondary analysis of data from participants randomized to the computer therapy group to investigate whether any demographic, clinical or intervention variables were associated with adherence to therapy practice. A sub-sample of the same participants took part in qualitative interviews exploring the factors perceived to influence the amount of aphasia computer therapy practice undertaken. Interviews were analyzed thematically. A convergence-coding matrix was used to triangulate the two sets of findings.

**Results:** Data from 85 participants randomized to the computer therapy group were included in the quantitative analyses. At a clinical level, a greater length of time post-stroke was associated with higher adherence to self-managed aphasia therapy practice on a computer. At an intervention level, length of computer therapy access and therapist time supporting the participant were associated with greater adherence to computer therapy practice. Interviews with 11 patients and 12 informal carers identified a multitude of factors perceived to influence engagement with tele-rehabilitation by people with aphasia. The factors grouped around three themes: capability to use the computer therapy, having the opportunity to practice (external influences and technological issues) and motivation (beliefs, goals and intentions vs. personality, emotions, habit and reinforcement). Triangulation demonstrated convergence for the finding that participants' practiced computer-based therapy exercises more when they received increased support from a speech and language therapist.

**Conclusion:** Clinicians delivering asynchronous tele-rehabilitation involving self-management of aphasia therapy practice on a computer should consider the factors found to be associated with engagement when deciding which patients may be suited to this option, as well as how they can be supported to optimize the amount of practice they engage in.

## Introduction

Aphasia is a communication disorder affecting speaking, listening, reading, and writing, often acquired after a stroke. Approximately one third of stroke survivors experience aphasia (1). Communication difficulties are known to reduce social participation in all aspects of life including domestic life, employment, and relationships with family and friends (2). Speech and language therapy for people with aphasia (PWA) aims to improve the ability to communicate and participate in everyday activities by directly addressing specific language impairments or by teaching strategies that compensate for the impairment. The most recent Cochrane Review of speech and language therapy for aphasia following stroke found evidence of the effectiveness of speech and language therapy compared to no treatment, but there is no evidence that one treatment was more effective than another (3). However, functional communication was found to benefit from therapy delivered at a high intensity, high dose, or over a long duration (3). There is ongoing debate about whether it is the intensity or total dose of therapy that enables it to be most effective; however, irrespective of favoring massed or distributed practice, there is agreement that more is better (4). It is also known that PWA can continue to recover long after they have had a stroke (5). However, due to resource constraints, limited speech and language therapy is provided beyond the first few months post-stroke in the United Kingdom (UK) (6). One potential solution to address the need to provide more therapy, over a longer period, is to provide greater opportunity for independent practice supported by technology.

Impairment-based speech and language therapy aims to promote neuroplasticity for language (7). Key principles underpinning neuroplasticity include: “use it and improve it,” specificity matters (the nature of the therapy dictates the nature of plasticity), salience matters (the training experience must be sufficiently salient to induce plasticity), intensity matters (sufficient training intensity is required to induce plasticity), and repetition matters (sufficient repetition is required to induce plasticity) (7). Deliberate practice facilitates the opportunity for repetition in order to improve skill acquisition (8). Carrying out that practice independently in their own home allows patients to have autonomy to carry out therapy whenever and wherever they chose, as well as increasing accessibility for those in rural and remote locations (9). Adherence to independent practice has been found to be facilitated by individualized therapy approaches, positive reinforcement, goal setting, feedback and problem solving (10, 11). Computerized speech and language therapy is thought to be an efficient solution to provide therapy in the longer term as it provides maximum opportunity for practice beyond that available through face-to-face interaction with a speech and language therapist (SLT) (12). Computerized home-based therapy for aphasia has been delivered through software programs (e.g., AphasiaScripts™) (13), specifically designed apps (e.g., TACTUS Language therapy^©^) (14), and generic apps (e.g., iBooks) (9). Asynchronous tele-rehabilitation is a service delivery model that enables patients to carry out independent practice of computer-based rehabilitation interventions in their own time, with monitoring and adaption of therapy exercises carried out asynchronously (15). The Big CACTUS trial was the first fully powered, randomized controlled trial to evaluate the clinical and cost-effectiveness of computer speech and language therapy for aphasia in the long-term post stroke (16, 17). In total, 287 participants were randomized to receive either the asynchronous tele-rehabilitation intervention, attention/activity control (puzzle book and phone calls) or usual care. The trial found that the intervention improved word finding, but this did not translate to improved conversation or quality of life. This suggests that the intervention is useful for improving the word-finding impairment. However, it might need adapting or to be used within a broader program of therapy to enable the benefit to translate to improved conversation.

Independent use of specialist aphasia software on a home computer can provide increased opportunity for the practice of rehabilitation exercises. The Big CACTUS trial confirmed that this form of tele-rehabilitation increased the amount of practice carried out over a 6-month period for a relatively low-cost of £733 per person, compared to £1,400 for the same amount of face-to-face therapy (17). Participants using the aphasia computer therapy practiced for a mean of 28 h (SD 25.6) compared with a mean of 3.8 h (SD 7.4) of usual care that was received by all participants over the same 6-month period. The large standard deviation demonstrates that there was significant variation in the amount of independent practice conducted (0–104.5 h), thus demonstrating that providing the opportunity for more therapy and the PWA actually carrying out more therapy do not necessarily equate. When rehabilitation exercises are delivered in their own home as a self-managed intervention, patients can decide to carry out as much or as little as they choose. This exploratory study sought to understand the reasons for variation in adherence to aphasia computer therapy in order to help clinicians target computer aphasia therapy at those who are most suited to this approach and identify how adherence might be optimized.

Carrying out regular independent practice of rehabilitation exercises requires behavior change (11). One of the most influential behavior change frameworks is the COM-B system, which proposes that it is a combination of capability, opportunity and motivation that determines whether or not a behavior will be enacted (18). The three causal elements of the COM-B framework can be further sub-divided. Capability is divided into physical and psychological capability, with physical capability referring to elements such as physical strength and skills, and psychological capability referring to having the psychological resources and skills to comprehend and perform the behavior (18). Opportunity is divided into the opportunity provided by the physical and social environment. The physical environment relates to aspects such as resources or location and social environment relates to aspects such as culture and language. Motivation is divided into reflective and automatic processes. Reflective processes require planning and evaluation, whereas automatic processes are based on basic drives, emotional responses, learnt associations and habit (18). The COM-B system was selected by the authors as a sensitizing framework to help interpret and structure the findings of this exploratory study.

The aim of this study was to explore the factors associated with adherence to self-managed aphasia computer therapy to help identify those individuals for whom this form of tele-rehabilitation may be best suited, and help clinicians optimize conditions to maximize engagement.

## Materials and Methods

### Design

A concurrent triangulation mixed methods approach was adopted to explore the factors associated with adherence to aphasia computer therapy (19). The qualitative exploration of adherence investigated a wide range of factors influencing patient's engagement with this tele-rehabilitation approach. The quantitative analysis was more limited as it could only include variables for which data had been collected within the Big CACTUS trial. Employing a concurrent triangulation approach ensured a greater breadth of possible factors were explored, whilst also capitalizing on the high quality data available from the Big CACTUS trial, and enabling cross-validation of the findings through triangulation of the two data-sets. The authors adopted a subtle realist stance, meaning that we only know reality from our own perspective of it (20), and therefore placed equal value on the qualitative and quantitative findings whilst acknowledging the limitations of both approaches. This study was conducted as part of the first authors PhD (21).

### Participants

The Big CACTUS trial recruited participants from National Health Service (NHS) speech and language therapy departments across the UK, aphasia support groups and advertisements displayed in public places. The inclusion criteria were: diagnosis of aphasia caused by a stroke, stroke having occurred 4 months prior to randomization, minimum 18 years of age, score between 5 and 43/48 on the Naming Objects sub-test of the Comprehensive Aphasia Test (CAT) (22), ability to perform a simple matching task in StepByStep with at least 50% accuracy to confirm they could use the software, and the ability to repeat at least 50% of words in a word repetition task. The exclusion criteria included the patient: requiring treatment in a language other than English, already using a computer therapy program to aid word finding, or having another pre-morbid speech and language disorder. A Consent Support Tool (23) was used to identify the support required to enable the patient to provide written informed consent or to identify those patients for whom a carer was required to provide written consent or a declaration of belief that they wished to participate. In addition to the trial inclusion criteria, participants also needed to have been randomized to receive the computer therapy intervention in the Big CACTUS trial and have practice data recorded for >3 months to be included in the secondary data analysis reported here.

A sample of those participants whose data were eligible for inclusion in the secondary data analysis were invited to take part in a qualitative interview. Patients with the most severe aphasia, who were unable to comprehend two key words in a sentence according to the Consent Support Tool (23), were excluded from the qualitative interviews as it was unlikely they could be supported sufficiently to understand the questions asked. However, in order to ensure that the views of patients with more severe aphasia were included we invited their carers to participate in a carer-only phone interview. All participants provided written informed consent.

Maximum variation sampling was used to identify a heterogeneous sample comprising participants who had carried out the highest and lowest amounts of practice in order to maximize the diversity of experience within the sample, as well as enabling the identification of important shared themes that cut across cases (24). Eligible Big CACTUS trial participants were listed according to the amount of practice carried out over the 6-month intervention period. Working inwards, those at the top and bottom of the list were invited to participate first to increase the heterogeneity of the sample. Sample size was determined by the concept of data saturation, which states that data collection stops when no new themes emerge from the data (25).

### Intervention

The tele-rehabilitation intervention, referred to as the StepByStep computer therapy approach for the NHS, comprises the specialist StepByStep^©^ aphasia software, with therapy set-up including personalization of vocabulary and tailoring of the exercises according to the individual's impairment provided by a SLT. In addition, on-going support is provided by a volunteer or therapy assistant to enable the PWA to carry out independent practice (16, 17). PWA were recommended to carry out 20–30 min of therapy per day over a 6-month period. This information was conveyed by the SLT and/or volunteer during a face-to-face demonstration of how to use the therapy. An information leaflet about the therapy was provided as a reminder. All support from SLTs or volunteers/assistants was provided via face-to-face contact, email, or telephone. The therapists were required to set up the computer therapy with tailored exercises and personally relevant practice items and provide a demonstration of how to use the therapy, but the number of contacts was not specified. Volunteers or therapy assistants were asked to provide 1 h of support per month. Support was recorded for 90% of participants (86/96) and one participant declined to receive the support offered. A median of 4 h 15 min of support was provided. As a pragmatic trial, there was no minimum amount of practice or number of sessions. Big CACTUS used available NHS resources and SLTs working in clinical practice to deliver the intervention (17). Additional detail about the delivery of the intervention within the Big CACTUS trial is available in the main results paper (16) and final report: https://www.ncbi.nlm.nih.gov/books/NBK556708/ (17).

### Quantitative Data Collection

All quantitative data informing the secondary data analysis were collected as part of the Big CACTUS trial (ISRCTN: 68798818). Data were collected by SLTs who visited the participants at their home to conduct language assessments and collect other necessary information. SLTs also documented the activities and amount of time they and the volunteer/therapy assistant spent supporting the participants, in order to be able to describe how the intervention was actually delivered (17).

The total amount, or dose, of computer therapy practice completed by the PWA was selected as the dependent variable for the purpose of this analysis as it is the measure of adherence most frequently described in the aphasia literature (26). Total practice time (hours) completed by participants was recorded on an electronic file (called a key file) by the StepByStep computer therapy program. The independent variables were divided into demographic, clinical, and intervention variables.

Demographic variables included: gender (male or female); age (≤ 55, 56–65, 66–75, ≥76 years old); presence of an informal carer (yes or no; an informal carer referred to a friend or family member); whether or not they had attended a support group in the 3 months prior to entering the trial; whether or not participants had internet access in their home and which site they were based at (recruiting Speech and Language Therapy department).

Clinical variables included: time post-stroke (years); number of strokes; type of aphasia (anomic, non-fluent, mixed non-fluent or fluent determined by therapists clinical judgement); evidence of apraxia of speech (yes/no based on therapists clinical judgement); severity of word-finding impairment (assessed by Naming Objects sub-test of the CAT) (22); comprehension ability (assessed by Comprehension of Spoken Sentences sub-test of the CAT); participants' own perception of communication related social participation and quality of life (assessed by Communication Outcome after Stroke (COAST) score) (27); and whether or not they had received care for communication difficulties in the 3 months prior to entering the trial.

Intervention variables included: the type of device used (tablet, laptop or desktop computer); who the device was owned by (owned by participant or loaned to participant); and how long the participant had access to the computer therapy software (number of days calculated based on date of provision, removal, and periods of inaccessibility recorded by the SLT) and therefore the opportunity to practice. Activity logs completed by the therapists recorded: (1) therapist time supporting the participant (minutes; this included providing technical support and monitoring the participants progress, directly or indirectly, and making adaptations to the therapy exercises; initial tailoring time not included); (2) therapy assistant/volunteer time supporting the participant (minutes; this included time spent setting-up/adjusting the computer or microphone, encouraging/motivating use of the computer therapy, providing assistance with using the software, and conversations to practice using the words they were learning with the software in context); and (3) therapist time spent with the therapy assistant/volunteer (minutes; including providing training, supporting the assistant/volunteer, providing technical support or monitoring a feedback form).

Demographic and clinical variables were collected prior to the participant's randomization in the Big CACTUS trial, whereas intervention variables were time-dependent co-variates having been collected after the participant had been randomized to the trial and at the same time as the adherence data was being collected. As such, temporality (cause proceeding effect), one of the Bradford-Hill criteria (28) for determining causation, cannot be assumed for the intervention variables, which is why the study can only report associations.

### Qualitative Data Collection

Participants were approached via an invitation letter and information sheet, in an accessible format for patient participants, followed by a telephone call from the first author. Interviews with patients took place face-to-face to enable the use of communication strategies. Carer-only interviews took place over the telephone. If both the patient and carer agreed to participate, the author sought to interview them separately, where possible, to allow the PWA to share their views without interruption. Informal carers who did not participate in the Big CACTUS study were asked to complete a short form collecting basic demographic information already collected about the informal carers participating in the Big CACTUS study (e.g., sex, date of birth, and relationship to PWA). The interviews were recorded using a digital recorder and transcribed verbatim. Transcriptions were checked for accuracy.

**Figure 1:**
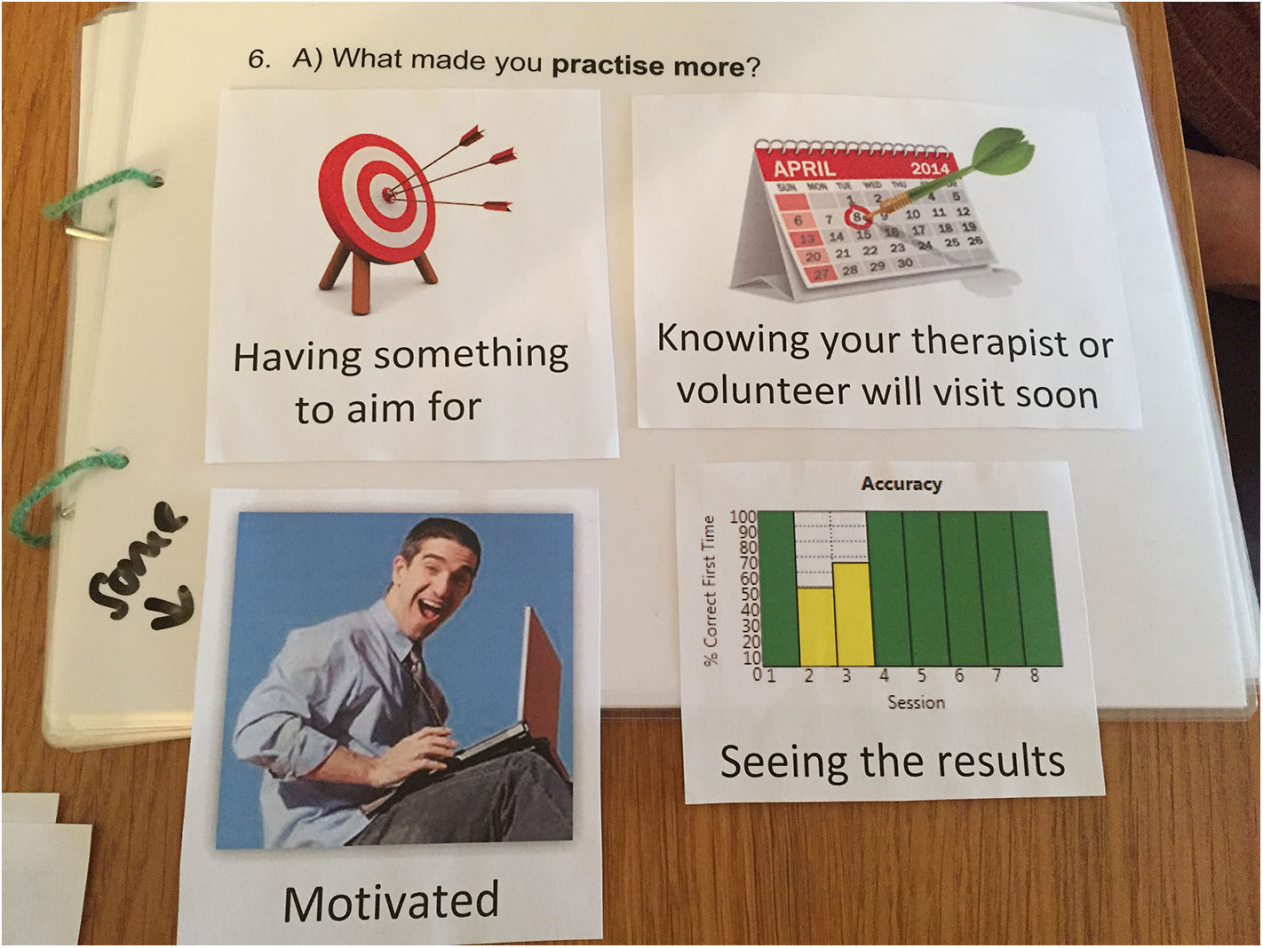
Example of picture selection cards used to facilitate response from participants with more severe communication impairment.

The development of the interview schedule was influenced by the COM-B system of behavior change (18). All interviews followed the same broad structure (see Table 1); however, the interview schedule for patient participants was tailored to their communication ability and supported with visual aids where necessary. The interview schedule was broken down into smaller more manageable questions categorized according to how grammatically and conceptually complex the question was: (1) all PWA were asked the most simple questions supported by visual prompts or cues (i.e., calendar) or a picture selection task to support participants to respond, (2) more complex questions/prompts were asked only of those PWA who were able to answer the first level questions with ease, and (3) the most complex questions were only asked of those PWA who answered the second level questions with ease. A picture selection task was only offered if a verbal response was not provided. The picture cards were developed on the basis of the findings from an earlier study exploring the acceptability of a similar intervention (29) and recommendations from the Big CACTUS Patient and Carer Advisory Group. Each card showed a key concept for the participant to select whether or not it reflected their perspective (see Figure 1). The first interview served as an internal pilot after which the author reflected on how the questions were asked and the answers that were forthcoming and made small changes to the wording of the questions as necessary.

**Table 1:** Summary of questions from the interview schedule.

**Interview schedule**
Can you tell me about how your communication problem affects your life?
How important is it to you that your communication problem improves?
Can you tell me about the speech therapy you have had before?
When [therapist name] told you about the computer therapy, what were your first thoughts?
When did you start using the computer therapy? When did you finish?
How many times a week did you practice? What made you practice more? What made you practice less?
How long did each practice session last? What made you practice for longer? What made you practice for less time?
How often and for how long did [therapist name] and [volunteer name] suggest you should practice?
Can you tell me about using the computer therapy?
How often did you see [volunteer name] and how long for? Can you tell me about your relationship with [volunteer name]? What did you do during the visits?
How often did you see [therapist name] and how long for? Can you tell me about your relationship with [therapist name]? How did [therapist name and volunteer name] feel about the computer therapy?
Did anyone else help you with the computer therapy? What help did they provide?

When a carer also participated, more conceptually challenging questions were addressed to them if the patient was not able to answer them. Carer-only interviews utilized an interview schedule covering the same concepts, but asking the questions from the perspective of the patient (e.g., how important is it to your husband that his communication problem improves?).

#### Data Analysis

Exploratory data analysis techniques were employed to investigate the relationships between the independent variables described above and adherence to practice. Analysis was carried out using SPSS v25. The first step was to establish which of the demographic, clinical, and intervention variables (i.e., independent variables) were associated with the dependent variable (total practice time). In order to achieve this, bivariate analyses were conducted using a correlation matrix for continuous variables, independent samples *t*-tests for binary categorical variables, and one-way ANOVA for categorical variables with two or more categories. All independent variables found to be significantly associated (*p* < 0.05) with total practice time were included in a multiple linear regression model. The model was adjusted for age and gender. The original model violated the assumption for homogeneity of the variance. Consequently, a sensitivity analysis was carried out using the square root of total practice time (the dependent variable), which significantly reduced the heteroscedasticity. The sensitivity analysis allowed the original model to be retained. Results of the original model and sensitivity analysis are reported.

Qualitative data were analyzed using thematic analysis, to identify from the patient and carer perspective the factors associated with adherence to aphasia computer therapy (30). The initial step of familiarization was achieved through repeated reading of all transcripts. In-depth paper and pen based coding of a transcript from one high and one low adhering participant resulted in the development of an initial coding framework. The initial coding framework was entered into NVivo 11 (31). During the process of coding subsequent transcripts in NVivo, more codes were added and others were merged, grouped or renamed. Once all transcripts had been coded by the first author (MH), the themes were reviewed by all authors and an external qualitative expert (SA). During the review process it was decided that the sensitizing frameworks underpinning the development of the interview schedule, would be drawn upon to support the data interpretation phase. Therefore, a two-step inductive and deductive analysis process was used in which an initial thematic analysis was mapped onto an established model (32). No tensions arose during the mapping process as the data had a good fit with the COM-B system. The lower level codes (sub-themes) were mainly unchanged; however, some were divided or combined where necessary to map onto the COM-B system. Higher-order theme names were re-defined and the findings were written up (30). In order to explore the similarities and differences between high and low adhering participants, a feature of the NVivo software was used to categorize the transcripts as cases with different attributes (e.g., high vs. low adhering participant) to enable patterns of response to be explored.

The triangulation approach combined the “following a thread” method, whereby each finding from one dataset is followed across to the other dataset (33), and applying a “convergence coding matrix,” in which the findings from each study are displayed together along with consideration of the extent to which the findings converge (34). Firstly, the factors associated with adherence identified in the qualitative interviews acted as the thread, which was followed across and searched for in the quantitative findings. The qualitative data continued to be grouped according to the COM-B system (18), thus enabling the quantitative data to be considered in light of this behavior change model. The convergence coding matrix was used to integrate the threads and convergence was coded using the following criteria: “Convergence: where findings directly agree; Complementary: findings offer complementary information on the same issue; Dissonance: findings appear to contradict one another; Silence: themes arising from one component study but not others” (34). Only quantitative variables found to be associated with adherence to aphasia computer therapy practice in the multivariate analysis were included in the triangulation.

### Ethical Approval

Ethical approval for the secondary analysis was obtained from Leeds West Research Ethics Committee (REC; 13/YH/0377) and the Scottish A REC (14/SS/0023). Separate approval for the qualitative interviews was obtained from the School of Health and Related Research REC at the University of Sheffield (008063).

## Results

### Participants in Secondary Data Analysis

The analysis included 85 of the 97 participants randomized to the intervention arm of the Big CACTUS study. Participants with no practice time data (*n* = 9) or partial practice time data (3 or more months data not recorded; *n* = 3) were excluded. The sample included 54% males (*n* = 46). Descriptive data for key variables are summarized in Table 2. Findings from bivariate analysis.

**Table 2:** Descriptive summary table of key variables.

**Variable**	**Mean**	**Standard deviation**
Total practice time (hours)	30.92	25.36
Age (years)	66.28	12.9
Time post-stroke (years)	2.43	3.03
Length of computer therapy access (days)	139.96	34.64
Therapy assistant/volunteer time supporting participant (minutes)	254.76	107.45
Therapist time supporting participant (minutes)	84.29	92.44
Severity of word-finding difficulties (CAT Naming Objects; maximum score = 48)	25.88	11.53

The results of the bivariate analysis are presented by variable group.

#### Demographic Variables

Male participants practiced more (*M* = 50.70 h, SD = 50.97 h) than female participants (*M* = 32.51 h, SD = 30.19 h) and an independent samples *t*-test determined the difference was statistically significant [t_(74.78)_ = 2.035, *p* = 0.045]. Age was grouped into four categories with a similar number of participants in each age group (≤ 55 *n* = 20; 56–65 *n* = 19; 66–75 *n* = 24; ≥76 *n* = 22). Those aged 56–65 practiced most (*M* = 60.43 h, SD = 42.71 h) and those aged 76 and over practiced least (*M* = 30.16 h, SD = 40.86 h). However, a one-way ANOVA demonstrated that there were no statistically significant differences between age group means [F_(3, 81)_ = 1.956, *p* = 0.127]. None of the other demographic variables were found to be significantly associated with total practice time. There was a trend for those with an informal carer to practice more (*M* = 46.17 h, SD = 46.39 h) than those without (*M* = 29.08 h, SD = 28.21 h), but an independent samples *t*-test established that this was not a statistically significant difference [*t*_(83)_ = −1.523, *p* = 0.131]. There was also no significant difference in the amount of practice carried out by those who attended support groups (*M* = 48.80 h, SD = 52.41 h) compared to those who did not (*M* = 36.34 h, SD = 32.46 h) determined by an independent *t*-test [t_(65.87)_ = −1.306, *p* = 0.196]. There was a slight trend toward those with internet access practicing more (*M* = 47.28 h, SD = 35.06 h) than those without internet access (*M* = 36.53 h, SD = 51.49 h); however, the results were not statistically significant as determined by an independent samples *t*-test [*t*_(83)_ = −1.14, *p* = 0.258]. A one-way ANOVA established that there was also no statistically significant difference in practice time between the different sites [F_(20, 64)_ = 0.872, *p* = 0.621].

#### Clinical Variables

There was a weak positive correlation between total computer therapy practice time and number of years post-stroke (*r* = 0.23, *n* = 85, *p* = 0.04). This was the only clinical variable found to have a statistically significant association and therefore the only clinical variable to go forward to the regression model. A bivariate correlation matrix established that all other continuously measured clinical variables had weak, negative non-statistically significant associations with total practice time: number of strokes (*r* = −0.18, *n* = 85, *p* = 0.099), severity of word-finding difficulty shown by CAT naming objects score (*r* = −0.052, *n* = 85. *p* = 0.634), comprehension of spoken sentences (*r* = −0.015. *n* = 85, *p* = 0.889), and PWA rated perception of communication rated using the COAST (*r* = −0.010, *n* = 82, *p* = 0.929). There was a trend toward those who had not received care in the last 3 months practicing more (*M* = 47.58 h, SD = 49.29 h) than those who had received care (*M* = 36.46 h, SD = 35.44 h); however, an independent samples *t*-test established this was not a statistically significant difference [*t*_(83)_ = 1.181, *p* = 0.241]. There was no statistically significant difference in total practice time between those with apraxia of speech (*M* = 40.67 h, SD = 37.90 h) and those without (*M* = 43.36 h, SD = 46.79 h) as shown by an independent samples *t*-test [*t*_(83)_ = 0.275, *p* = 0.784]. A one-way ANOVA found no statistically significant difference in practice time between participants with different types of aphasia [F_(3, 81)_ = 0.277, *p* = 0.842].

#### Intervention Variables

Total practice time was found to be positively correlated with length of computer therapy access (*r* = 0.433, *N* = 85, *p* = 0.00), therapist time spent supporting participants (*r* = 0.242, *N* = 85, *p* = 0.026) and therapy assistant/volunteer session time spent supporting participants (*r* = 0.237, *N* = 79, *p* = 0.035). The amount of time the therapist spent with the therapy assistant or volunteer showed no linear relationship with the total amount of practice and was not statistically significant (*r* = 0.069, *n* = 80, *p* = 0.545). There was a trend toward more practice being carried out by those participants who were practicing on their own device (*M* = 50.44 h, SD = 34.76 h) rather than a device loaned to them (*M* = 38.79 h, SD = 46.58 h); however, an independent samples *t*-test demonstrated that the difference was not statistically significant [*t*_(83)_ = 1.141, *p* = 0.257]. Participants could practice on three types of device: the majority used a laptop (*N* = 70, *M* = 41.09 h, SD = 39.70 h), some used a tablet (*N* = 12, *M* = 52.83 h, SD = 65.05 h), and a small number used a desktop computer (*N* = 3, *M* = 29.76 h, SD = 25.42 h). Whilst there was a trend for participants using the most portable device (tablet) to practice most and the least portable device (desktop computer) to practice least, the number of participants in the three groups was unequal and a one-way ANOVA showed that the difference between the groups was not statistically significant [F_(2, 82)_ = 0.498, *p* = 0.609].

### Findings From Multivariate Analysis

Multivariate linear regression was carried out to investigate the relationship between practice time (hours) and time post-stroke (years), length of computer therapy access (days), therapist time supporting participant (minutes), and therapy assistant/volunteer time supporting participant (minutes). The model included data from 79 of the 85 participants due to missing data. The analysis was controlled for age and gender. There was a statistically significant relationship between practice time and length of time post-stroke (*p* = 0.038), computer therapy access (*p* = 0.003), and therapist time supporting participant (*p* = 0.043). For each additional year post-stroke, there was a 3.018 h (3 h 1 min) increase in practice time (see Table 3). For each additional day of computer therapy access, there was a 0.124 h (7 min) increase in practice time. Furthermore, for each additional minute the therapist spent providing support (including technical support and monitoring/adapting exercises) to the participant the total practice time increased by 0.098 h (6 min). Gender, previously found to be associated with practice time in the bivariate analysis, no longer demonstrated a statistically significant association with practice time in the multivariate analysis (*p* = 0.110). The relationship between practice time and therapy assistant/volunteer time supporting the participant (*p* = 0.233) was also not statistically significant.

**Table 3:** Regression coefficients and *p*-values for the original and square root multiple linear regression models.

**Variable**	**Original model**			**Square root model**		
	**Coefficient**	***p*-value**	**Confidence interval**	**Coefficient**	***p*-value**	**Confidence interval**
Time post-stroke (years)	3.018^*^	0.038	0.170–5.866	0.241^*^	0.028	0.027–0.455
Length of computer therapy access (days)	0.124^*^	0.003	0.043–0.204	0.007^*^	0.029	0.001–0.013
Therapy assistant/ volunteer time supporting participant (minutes)	0.054	0.233	−0.036 to 0.144	0.007^*^	0.041	0–0.014
Therapist time supporting participant (minutes)	0.098^*^	0.043	0.003–0.193	0.009^*^	0.020	0.001–0.016
Gender	−14.453	0.110	−32.233 to 3.327	−0.635	0.347	−1.971 to 0.701
Age (years)	2.400	0.556	−5.686 to 10.485	−0.071	0.818	−0.678 to 0.537

* *Significant at 5% level*.

Therapy assistant/volunteer time supporting the participant would have been removed from the model due to non-significance; however, it was retained because it was identified to be significant in the sensitivity analysis. A scatterplot of standardized predicted values vs. standardized residuals indicated that the data did not meet the assumption of homoscedasticity. The sensitivity analysis, using a square root model, allowed for the assumption of homoscedasticity to be met, thus confirming the findings of the original model. One notable difference between the two models was that therapy assistant/volunteer time supporting the participants, which was not significantly associated with practice time in the original regression model, was found to be statistically significant in the square root model (see Table 3). This will be taken into consideration in the interpretation of results in the discussion.

The *R*^2^ value for the original model was 0.29, so 29% of the variation in practice time can be explained by the model containing age, gender, time post-stroke, length of computer therapy access, therapist time supporting the participant and therapy assistant/volunteer time supporting the participant.

### Participants in Qualitative Interviews

In total, 14 interviews were conducted with 23 participants, including 11 PWA and 12 informal carers. The mean interview length was 68 min (ranging from 24 to 103 min). The mean time between the end of the 6-month intervention period and participation in the interview was 247 days (SD = 113.21). Nine interviews included the PWA and carer, three were carer-only interviews and two were PWA-only interviews. All carer-only interviews were conducted with the carers of low adhering patients. Of the PWA participating, or described in a carer-only interview, the mean age was 65 years old (ranging from 48 to 85) and 10/14 were male. The mean practice time for high adhering (HA) participants was 67 h 21 min, and for low adhering (LA) participants, 13 h 13 min.

Both the low and high adhering groups included participants whose aphasia was classified as mild (score 65–90%), moderate (score 35–64%) and severe (score 10–34%) on the CAT naming objects assessment (22). With the exception of one participant who died prior to the 6-month outcome assessment and one participant who did not carry out any independent practice, all participants showed some improvement on the personal vocabulary naming test, in which they had to name the items they were practicing on the StepByStep software. The mean age of the carers was 61 years old (ranging from 46 to 76) and one was male. The relationship of the carers to the PWA included: eight wives, one partner, one mother, one daughter and one son.

### Findings From Qualitative Interview

The factors grouped around three themes influenced by the COM-B system (18): capability to use the computer therapy, having the opportunity to practice, and motivation. The findings are summarized in Table 4.

**Table 4:** Factors associated with adherence to aphasia computer therapy categorized into themes using the COM-B system.

**Capability**	**Opportunity**	**Motivation**
Physical	Physical	Reflective
• ↕ Ability to use computer therapy software • ↑ Assistants/volunteers help PWA to develop the skills required to use the computer therapy	• Computer therapy software problems (↓issues with voice recognition; ↓stability of the software; ↑stability of the software was improved via software updates) • ↓Computer hardware problems • Features of the software that facilitated more practice (↑ personalization of vocabulary; ↑therapy in home environment; ↑ independence HA only) • Barriers to practice (↓periods of illness; ↓other commitments; ↓engaging in alternative therapeutic activities) • Availability of support (↑more input from supporters; ↑informal carers of participants who could not use computer therapy independently)	• Beliefs about capability (↓capability concerns often based on lack of prior computer experience; ↑high self-efficacy HA only) • Beliefs about consequences (↑expectation of anticipated outcome influenced by supporters; ↓pessimism) • Goals (↑distal goal associated with regular practice; ↑proximal goal associated with longer practice session; ↓mismatch between personal goal and intended outcome of computer therapy) • Stability of intentions (↓ LA described decline over time) • ↓carer more motivated than PWA
Psychological	Social	Automatic
• ↕ Knowledge of recommended practice time • ↕Understanding/knowledge of own condition • Cognitive impairment and fatigue (↓forgetting; ↓concentration problems; ↓fatigue; ↑strategies to overcome e.g., practice certain times of day)	• ↑ External support (importance of input from SLT or volunteer/assistant) • ↑ Social pressure (caused by impending visit from supporter)	• Emotion (↓low mood or negative attitude on given day) • Personality (↑determined/perfectionist) • Habit (↑routine pattern of practice) • Reinforcement (↑feedback about practice time; • ↕feedback about performance)

*↓, factor associated with less practice; ↑, factor associated with more practice; ↕, factor associated with both more and less practice*.

#### Capability to Use the Computer Therapy

Psychological capability was discussed in relation to participants' knowledge of the intervention, understanding of their own condition and the impact their cognitive impairment had on the amount of practice conducted. Participants' recall of the recommended amount of practice they were expected to carry out was variable. Most participants recalled the recommended duration specified in the treatment manual (20–30 min), but the recommended frequency of practice recalled by participants varied from “*everyday*” to a “*few times a week”* with no clustering of responses around the recommendation that practice should be carried out every day as specified in the treatment manual. Some participants described relatively strict practice guidelines from therapists; however, low adherers more often felt the decision regarding whether or not to practice was based on personal preferences.

R15/01 carer: She said obviously don't let it take over your life but she really sort of left it to us to work out fitting into the lifestyle as to how much he should or shouldn't do. (LA)

Aphasia is a complex condition and participants had varying levels of understanding or knowledge about their own condition. The lowest adhering participant, who scored <50% on the naming assessment at baseline and 6 months, reported that he could name all of the vocabulary available to practice on the computer, therefore demonstrating a lack of awareness, or possibly denial, of their own communication impairment, which might explain their lack of motivation to practice. Some other low adhering participants expressed similar thoughts. In contrast, having more knowledge and understanding of aphasia, as well as having more insight into the impact their communication impairment had on their lives was a motivating factor for those participants who were able to describe how their impairment affected their everyday life.

R16/07: Yeah. It wasn't making any good to me anyway, cause I knew exactly what they were cause they're already in there. I could say all of these things. (LA)

R10/02: I realise my language was letting me down see. (HA)

Some perceived practice to have been limited by stroke-related cognitive impairments, such as difficulties with memory, concentration, and fatigue, and described strategies they had identified to overcome the impact of cognitive impairment, such as practicing at a time when they are most alert or moving to a quieter area of the house.

R13/21 carer: She can't concentrate on more than one thing at once, if you know what I mean and children sometimes can be, you know, in the background and not even being noisy, but you are conscious of them, aren't you? (LA)

Another carer described how impaired cognitive functioning prevented independent practice meaning someone had to be available to help him to use the computer therapy, thus demonstrating an association between reduced capability and reduced opportunity for practice.

R01/40 carer: He couldn't quite work everything out on his own so it was always with somebody. (LA)

Low-adhering participants with less computer experience also described challenges related to their ability to physically navigate the computer therapy. Participants felt the assistants/volunteers played a vital role in overcoming this barrier.

R13/21 carer: I think just sorting out the programme, you know, how to get from one bit to the other and sometimes, you know, it was a bit difficult but I think when [assistant] came she sort of, you know, showed her how to get from one bit to the other. (LA)

#### Having the Opportunity to Practice

The physical aspects of opportunity predominantly related to the StepByStep software and the hardware to run it on, as well as the support provided by SLTs/volunteers/therapy assistants. Computer therapy software problems were described as a significant barrier to practice. In particular, problems were encountered with the voice recognition component of the software, which provided feedback on the participant's performance. Whilst nearly all of the participants described the issues with voice recognition as frustrating, some participants continued to practice with work arounds suggested by therapists, including being told to skip that aspect of the computer therapy or in some cases the therapist turned off the voice recognition component. However, for some participants, particularly those who perceived a need for reinforcement, skipping the voice recognition step was not a satisfactory solution and resulted in reduced practice. The other issues with the software reported by participants related to the stability of the software including the software crashing and not being able to move between the different sections or exercises within the software. Some of the issues with the stability of the software related to the fact that the software was an early release of version 5 of the StepByStep software. Several updates were available during the time participants were using the software, and participants described performance improving after updates.

R10/02 carer: It was taking a while for the voice recognition on the microphone to log with the computer, you know, and the computer go ‘ping', tick, you know. Um, so [PWA] would, would have to say it two, three, four times and that was then stopping the computer and that, that became quite, er, frustrating for him and then sometimes it, it would be just seize up. (HA)

R10/37 carer: I don't think it was perfect to start with. I think it's got better and we've had a couple of updates on it since. (HA)

A smaller number of participants had computer hardware problems which prevented access to the software. Most of the problems described were the result of using outdated (e.g., slow operating system or operating system not compatible with software) or unfamiliar (e.g., participant having to learn to navigate a new computer system) hardware. The process of determining whether the cause of the problems lay with the technology itself or the way in which the participant was using it demonstrated links between having the opportunity to use the computer therapy and the participants' actual or perceived capability to do so.

Interviewer: So my next question was what made her practice less?

R03/39 carer: I think cause of the problems with the laptop. (LA)

R03/39 carer: So I never knew whether that was my fault-, whether it was our fault or the computers fault. I mean, I know she did swap it over at one time and I said, ‘can you not get us a new one?' ‘No we're not allowed new ones', she said, ‘it's all old ones'. (LA)

Participants described several features of the software that enabled more practice, including: the ability to personalize the vocabulary, being able to carry out the therapy in their home environment and for high adhering participants the opportunity to engage in independent activity.

R15/37 carer: That was something that made you practice more because they were your words and not just on a computer they were the words you wanted to say. (HA)

R10/37: Oh yes. I get on with it, away-, leave me on my own. (HA)

Barriers to practice identified by participants included: periods of illness, having other commitments (holidays, other appointments, receiving physical care or caring for others) and engaging in alternative therapeutic activities (word games, naming picture cards or educational computer programs designed for children). Carers of low adhering participants described encouraging the participant to engage in these alternative activities when the PWA did not want to use a computer.

R11/03: The only time days off was cause I was going on a cruise. (HA)

R19/19 carer: We done a few little things ourselves off the internet, like we'd have stuff like, for want of a better word, like food and transport and animals and what-have-you. So if she didn't want to go on the computer, which we always tried to get her on the computer at least a half hour every day, we bring out like our own little flip sheets. (LA)

Almost all participants, including those with limited expressive communication (using the picture selection task), expressed that having help and support available from a therapy assistant/volunteer enabled more practice. Some participants, particularly those with more significant communication impairment expressed a need for more help and support. One carer participant reported that their spouse received minimal input from a volunteer/assistant and felt that they would have been more motivated to practice if regular support had been available.

R15/01 carer: It would probably have kept his motivation a little higher in the respect that people would come round, not just to sort of click a memory stick in and take out a reading and see what's been done. I think if someone had come and sat with him, you know, maybe every six weeks, or a month or something like that, you know, somebody who's a professional, not me. (LA)

As well as having the physical opportunity to carry out aphasia computer therapy practice, participants also described the social opportunity afforded by their interactions with others. Several carer participants described the importance of having external support from a speech and language therapist, assistant/volunteer or more removed family member. It was perceived, particularly by the primary informal carers, that support from an external agent was more beneficial and allowed the PWA to engage more fully.

R16/04 carer: Yeah the prompting and the people that aren't me telling him because he doesn't listen to me in the same way and I understand, why would he? But he is better if it is people outside, it would have been better. (HA)

The added benefit of an external supporter was that their visits created a social pressure to carry out more practice. Participants described upcoming visits triggering a sense of obligation to practice and the desire to please the supporter.

R10/37: Erm. We did it because we'd been asked to do it. (HA)

#### Motivation: Beliefs, Goals, and Intentions vs. Personality, Emotions, Habit, and Reinforcement

In addition to participant's actual capability to engage with asynchronous tele-rehabilitation, they also described beliefs about their own capability to perform the therapy (i.e., self-efficacy). Capability concerns were primarily expressed by low adhering participants based on their lack of prior computer experience. Contrastingly, some high adhering participants described a strong belief in their own ability to engage in computer therapy prior to commencing therapy irrespective of their familiarity with computers, suggesting their beliefs might actually be better predictors of engagement than prior computer experience.

R16/07: Well when they said that I could use a computer I thought, ‘I won't be able to do that, how am I going to do that?' […] I mean-, we've never had a computer. (LA)

R11/03: An' I thought I'm not too much into computers, but it's easy innit? Honestly it's easy, just click it and job done. So I thought perhaps I can handle that for half an hour, I can handle that. (HA)

As well as belief in their own capability, participants also described their beliefs about the consequences of the intervention. Participant's descriptions of carrying out continued regular practice responded to an underlying expectation that regular practice would result in an improvement in their performance and overall recovery from their aphasia. The expectation of improvement may have resulted from the fact that most participants perceived that the supporters believed in the effectiveness of the aphasia computer therapy and thought it would be a good opportunity for the PWA.

R06/01: I got the impression she [therapist] believed in it, I think, yeah. Cause if I hadn't got that impression I wouldn't have continued with it, so yeah, yeah. (HA)

Contrastingly, a carer of one of the low adhering PWA described the participant's pessimism and lack of belief that the intervention could produce a beneficial outcome.

R16/07 carer: I think you didn't give it a chance, but you just said, ‘what's this doing to help me?' (LA)

High adhering participants and their carers, who typically had a good understanding of their impairment more frequently described their distal goals (i.e., long-term) in terms of what they hoped to achieve from carrying out regular practice. Proximal goals (i.e., short-term) were perceived to motivate longer individual practice sessions. Some participants described goals having been set for them by SLT or assistants/volunteers which they also found to be motivating, particularly when they were combined with feedback from the computer therapy software.

R11/03: She [the assistant] would go through a few of them and see how I was doing and at one stage [therapist] said to her if I want to move on I've gotta get above 90%. And I was getting almost 90% for most of them and that's sort of inspired me to crack on with it. (HA)

For one low adhering participant there appeared to be a mismatch between the goal of the patient and carer (improved conversation) and the perceived goal of the intervention (naming more words).

R16/07 carer: He could say donkey, horse and things like that and name them. But to me that is not what-, he needed conversation, not particular things you need. (LA)

Participants, particularly carers of low adhering patients, described changes in the stability of their intention to practice. Where a change in motivational readiness was described it was typically a decline in practice over time as initial excitement or interest reduced combined with other influences, such as lifestyle changes or a reduced belief in the consequences.

R15/01 carer: I think it's like a lot of things in life, isn't it, you know, you start off, you are very highly motivated and then when you are kind of left to your own devices, it starts to peter out. (LA)

Some carers of low adhering participants described different levels of intent between themselves and the patient, with the carer encouraging more practice. The social pressure carers applied appeared to result in individual practice sessions, rather than sustained practice. For one patient-carer dyad the mismatch in intent resulted in conflict, potentially indicating the importance of the PWA expressing their own interest in engaging with aphasia computer therapy.

R19/19 carer: Now and again I think she found it in herself like, ‘oh I don't want to do this today', and it would cause-, well we might have a bit of a row. I'd say, ‘come on mam you've gotta do this, you've gotta', and she would, ‘no', she didn't want to know. (LA)

Automatic motivational factors were also described, such as emotions, personality, habit and reinforcement. Carer participants, particularly those of low adhering patients, perceived that the emotions experienced by the PWA, particularly their mood and attitude on each individual day, played a significant role in their decision to practice. High adhering participants described personality traits, such as determination and perfectionism, that positively influenced their engagement.

R19/19 carer: But when she had a good day you could see she was happier and she was just ‘bom', she'd go through [the exercises] no problem. (LA)

R06/01 carer: He's so determined and so-, if he sets his mind to something he wants to do it and wants to do it really well. (HA)

Most of the high adhering participants described developing a routine pattern of practice which resulted in a habit being formed thus increasing the automaticity of the behavior. The routine either involved doing it every day at the same time or having a regular trigger, for example PWA's spouse watching a television drama in the evening or imitating the working week.

R10/37: I just thought I was doing a job and I just did it like a job. So I did it five days, seven days and then I'm back. (HA)

The StepByStep software provides two types of reinforcement: feedback about the amount of practice time completed and feedback on word-finding ability. Participants found feedback about practice time on the color coded calendar (yellow = some practice, but <20 min; green = more than 20 min practice) motivated them to practice for longer.

R11/03: If it comes down and it's got like a yellow thing that's no good, that's about twenty minutes or so, that's no good I've got to get a green. So you've got to be at least half an hour, maybe a little bit over the top for it to actually transmit. (HA)

Participants valued the feedback the StepByStep software provided on naming performance when the voice recognition function was available and on the spelling tasks in the “using writing to cue naming” exercise. In confirmatory responses during the picture selection task, participants felt seeing the results, trying to do better than last time and trying to achieve 100% were factors that motivated them to carry out more practice. Feedback on performance was perceived to be one reason why computer therapy could be more motivating than paper-based exercises provided by SLTs.

R10/02 Carer: The computer was something very real that he could see and, and perform against, or with on a day-by-day basis and that really suited [PWA]'s, um, learning, or the way he, you know, works. […] He could see his performance, he could see he was making improvement. (HA)

### Triangulation Findings

The quantitative data yielded four factors potentially associated with adherence to asynchronous tele-rehabilitation for aphasia, compared to 21 factors identified in the qualitative data. The more comprehensive qualitative data was used as the basis of the “following a thread” method. The convergence coding matrix is presented in Table 5.

**Table 5:** Convergence coding matrix.

**Elements from the COM-B system used to frame integration**	**Factors associated with adherence identified through interview data**	**Factors associated with adherence identified through secondary data analysis of Big CACTUS trial data**	**Convergence assessment (34)**
Physical capability	↕ Ability to use computer therapy software	N/A	Silence
	↑ Assistants/volunteers helped PWA to develop the skills required to use the computer therapy	↑Assistant/volunteer spending more time supporting the participant	Complementary
Psychological capability	↕ Knowledge of recommended practice time	N/A	Silence
	↕Understanding/knowledge of own condition	N/A	Silence
	Cognitive impairment and fatigue (↓forgetting; ↓concentration problems; ↓fatigue; ↑strategies to overcome)	↑Longer length of time post stroke	Complementary
Physical opportunity	Features of the software that facilitated more practice (↑ personalization of vocabulary; ↑therapy in home environment; ↑ independence HA only)	N/A	Silence
	Barriers to practice (↓periods of illness; ↓other commitments; ↓engaging in alternative therapeutic activities)	↑Longer length of time post stroke	Complementary
	Computer therapy software problems (↓issues with voice recognition; ↓stability of the software; ↑stability of the software was improved via software updates) ↓Computer hardware problems	↑Computer therapy available for longer	Complementary
	Availability of support (↑more input from supporters; ↑informal carers of participants who could not use computer therapy independently)	↑Therapist spending more time supporting the participant ↑Assistant/volunteer spending more time supporting the participant	Convergence
Social opportunity	↑ External support (importance of input from SLT or volunteer/ assistant)	↑Therapist spending more time supporting the participant	Complementary
	↑ Social pressure (caused by impending visit from supporter)	↑Assistant/volunteer spending more time supporting the participant	
Reflective motivation	Beliefs about capability	N/A	Silence
	Beliefs about consequences	N/A	Silence
	Goals	N/A	Silence
	Stability of intentions	N/A	Silence
	Differing intention between PWA and carer	N/A	Silence
Automatic motivation	Emotion	N/A	Silence
	Personality	N/A	Silence
	Habit	N/A	Silence
	Reinforcement	N/A	Silence

*↓, factor associated with less practice; ↑, factor associated with more practice; ↕, factor associated both with more and less practice*.

Qualitative and quantitative findings about patient's capability to use the computer therapy were either complementary or there was silence in the quantitative data. The vital role assistants/volunteers played in enabling participants to develop the skills required to be physically capable of using the tele-rehabilitation exercises was recognized in the qualitative interviews. This complemented the quantitative finding that more assistant/volunteer support was associated with greater adherence in the square root analysis, by providing a possible explanation for why more support might lead to greater adherence. The psychological capability of participants to adhere to regular computer therapy practice was perceived to have been impeded by cognitive impairment and fatigue, both of which can improve over time, which could potentially provide an explanation for the quantitative finding that people were more adherent the more time had passed since their stroke.

Findings relating to the patient's opportunity to engage in practice were either complementary, silent and in one instance, the qualitative and quantitative findings converged. In itself, the passing of time since a patient's stroke is unlikely to have resulted in greater adherence. It is more likely a proxy for recovery, as described above, or lifestyle changes that have taken place over time. Some of the barriers to practice which reduced the participants' physical opportunity to practice included having other commitments or engaging in other therapeutic activities. It is possible that participants might have fewer other commitments or less opportunity to engage in alternative therapeutic activities, the more time that has elapsed since their stroke, thus reducing some of the barriers identified in the qualitative interviews. Another factor perceived to reduce participant's physical opportunity to practice was problems with the computer therapy software and computer hardware, both of which resulted in the computer therapy being available to the participant for less time. This provides a possible explanation for why the length of time the computer therapy was available to participants was significantly associated with adherence in the quantitative findings. The broad consensus from the qualitative data was that high adhering participants perceived greater availability of support (from both therapists and assistants/volunteers) enabling more practice. Some low adherers who perceived they did not have enough support felt they would have been able to practice more if they had increased support. This finding demonstrates convergence with the quantitative findings that the therapist (and possibly the assistant/volunteer) spending more time supporting the participant was significantly associated with greater adherence. The same quantitative finding that more support from therapists and assistants/volunteers facilitated more practice was also complementary in terms of the qualitative finding that external support was perceived to be more beneficial due to the social pressure created by that support not being provided by someone well known to them. This demonstrates the importance of providing “outside” support rather than relying on family carer support.

None of the quantitative data collected corresponded with any of the factors relating to motivation from the qualitative dataset meaning there was silence across all factors associated with motivation.

## Discussion

This mixed methods study explored the factors associated with adherence to self-managed aphasia therapy on a computer. Quantitative findings suggested greater adherence is associated with more time having elapsed since the patient's stroke, the patient having access to the computer therapy for longer and the therapist spending more time supporting the participant. Findings from the qualitative interviews were grouped into three themes informed by the COM-B system (18): capability, opportunity and motivation. Factors identified as being associated with the patient's capability to adhere to aphasia computer therapy practice included: cognitive impairment, fatigue, level of understanding of their own condition, knowledge of the intervention, and therapy assistant/volunteer's help to develop the skills required to use the computer therapy. Factors that positively influenced PWA's opportunity to practice included receiving more support from therapists and volunteers/assistants and specific features of the software used (home-based therapy and personalization). Conversely, factors that negatively influenced PWA's opportunity to practice included software and hardware problems, illness, and having other commitments. Motivational factors that influenced adherence comprised PWA's beliefs about their own capability, beliefs about the likelihood of improvement, stability of intentions, reinforcement (via feedback from software), and habit. Triangulation demonstrated several complementary findings in which the qualitative data provided possible explanation for the quantitative findings, but also a lot of silence as the quantitative data were collected for the purposes of the Big CACTUS trial. The triangulation also identified convergence between the two datasets for the finding that people were more engaged with practicing their asynchronous tele-rehabilitation exercises when they received more support from SLTs, providing cross-validation for this finding.

Qualitative interviews highlighted the benefit of on-going support from both SLTs and volunteers/assistants, including the social pressure exerted by external support, the importance of support to enable the patient to develop skills to use the computer therapy and the absence of support being suggested as a reason for low adherence. The amount of time spent by the therapist supporting, monitoring, and adapting the software was found to be predictive of adherence to aphasia computer therapy. Similar findings were identified in a study investigating adherence to home-based exercise programs for neck and low back pain in which patients who received frequent supervision of their exercises had higher levels of adherence (35). Whilst only identified in the square root model and thus to be interpreted with caution, the finding that assistant/volunteer support was associated with more practice time echoed findings from the CACTUS pilot study. Most of the participants in the pilot study (3/4) who did not carry out the recommended amount of practice had not received contact from volunteers (36). These findings are indicative of the beneficial impact on-going support and monitoring can have on patient adherence to aphasia computer therapy. This need for support needs to be taken into consideration by those recommending self-managed therapies as a low-cost option. However, the Big CACTUS trial confirmed that the StepByStep computer therapy approach for the NHS is still a low cost option compared to providing the same amount of therapy face-to-face, despite the need for SLT/assistant time in set-up, personalization, and support. The Big CACTUS trial also identified that the intervention was more likely to be cost-effective for patients with mild-moderate word finding difficulties than those with severe word finding difficulties (17).

One of the reasons for attempting to deliver aphasia therapy in a self-managed computerized form is to enable the provision of speech and language therapy in the longer term post-stroke as evidence has demonstrated the effectiveness of such provision (>6 months) (5). Despite evidence of effectiveness, it has been established that PWA in the UK receive less face-to-face SLT the more time that has passed since their stroke (6). The finding that length of time post stroke was associated with greater adherence suggests that the intervention is possibly better suited to those in the longer-term post stroke. In the initial aftermath of a stroke there is a lot of change both mentally, in terms of psychological adjustment, and physically, in terms of receiving other care and rehabilitation interventions. The increased tolerance/adherence could be due to the PWA having more time to focus on therapy or due to a greater understanding of their condition.

Engaging with asynchronous tele-rehabilitation, such as self-managed aphasia computer therapy, requires behavior change. The COM-B system informed the development of the interview schedule and subsequently provided a useful structure upon which to frame the findings from the qualitative interviews and subsequently the convergence coding matrix. COM-B forms the hub of the Behaviour Change Wheel, which also identifies intervention functions that can be incorporated or adapted to enable an intervention to effect behavior change (18). The functions relevant to this tele-rehabilitation intervention include: incentivization, education, training, environmental restructuring, and enablement. We have considered how the functions of the intervention could be adapted to increase the amount of practice carried out by PWA based on the findings of this study. Feedback provided by the aphasia therapy software provided an incentive to practice. Auditory feedback and knowledge of performance, as were provided by the StepByStep software, have been found to be associated with improved performance in stroke rehabilitation more broadly (37). Visual feedback on the amount of practice carried out motivated more practice; however, feedback on performance was only perceived to be motivating when the feedback was accurate. When the voice recognition failed to recognize correct responses, it resulted in frustration and reduced motivation to practice. Improvement of the voice recognition by the software developers would increase the reliability of this incentive thus increasing automatic motivation. The software developers have already started to address this issue, and improvements to the voice recognition have been included in software updates.

The only finding from the triangulation for which qualitative and quantitative data converged was the finding that more support was associated with more practice. Therapists and assistants/volunteers were responsible for training and educating participants about the intervention. Having more time to provide training and education would have the potential to target multiple elements of the COM-B behavior change system through developing the skills to use the computer therapy (i.e., physical capability), increasing knowledge of recommended practice time (i.e., psychological capability), and knowledge about the potential consequences of the intervention (i.e., reflective motivation). Therefore, a clinical recommendation would be to ensure sufficient therapist and assistant/volunteer time is available and intervention development work could include amending training materials to reflect the importance of helping PWA to understand the intervention, the potential benefits and develop the necessary skills to use the computer therapy. A study by Cherney also highlighted the importance of SLT oversight when evaluating the delivery of an asynchronous tele-rehabilitation intervention called Web-ORLA™, although the support in that study was delivered by a virtual therapist rather than home visits (38). In addition, a recent review found that social support and therapist guidance were key factors contributing to adherence to home based exercise practice (39). Environmental restructuring (e.g., extended loan periods/installing software on the participant's own device) and enablement (prompt input from therapists and assistants/volunteers and software developers to reduce the impact of software and hardware problems) are both functions that could be targeted to increase the physical opportunity to practice. Furthermore, targeting the therapy to those participants who had their stroke a longer time ago might enable more therapy by reducing potential barriers highlighted by some participants in the qualitative interviews such as engaging in other therapies or having too many other commitments.

## Clinical Implications

Clinicians delivering computer therapy should consider the capability of participants to use the computer therapy, including factors such as, cognitive impairment, understanding of their condition, knowledge of the intervention and the role supporters can play in skill development. Furthermore, PWA might have more capability and opportunity to use the computer therapy once more time has passed since their stroke. Clinicians play a vital role in providing the opportunity to practice, which was as much about the need for support from SLTs and volunteers/assistants as it was about ensuring the computer therapy was available for a long period. One of the key roles of the supporters was to provide technical support, which was required to overcome the technological issues with the computer therapy highlighted in this study. Additionally, specific features of therapy software should be considered in relation to the opportunity they provide for practice, with the ability to use the software in their own home and the personalization of practice vocabulary being highlighted as beneficial for the StepByStep software. Clinicians should also consider how the motivation of the PWA might influence their decision to practice, with potentially modifiable motivational factors including creating shared goals and beliefs about the computer therapy, ensuring feedback on performance from the computer therapy is accurate as possible (or not selecting options that do not provide adequate feedback), and helping the PWA to develop a practice routine.

## Future Research

We know that higher doses of aphasia therapy have been found to be more effective (3) and more therapy is being delivered remotely, particularly in response to COVID-19 (40). Consequently, the need to understand more about who can best engage with computer based therapies and how we can support them to adhere to technology based therapies has never been so great. More research is needed to explore the factors associated with adherence to other computer based therapies to establish whether our findings are more widely applicable. In addition, the qualitative interviews highlighted the importance of motivational factors; however, more research is needed to find quantitative measures of motivation applicable for use with people with aphasia. The findings from this research will also feed into the iterative process of intervention development of the StepByStep approach and future research will be required to evaluate any changes made on the basis of these findings.

## Strengths and Limitations

This novel study is the first in depth exploration of adherence to self-managed aphasia computer therapy. The mixed methods approach found no divergent findings, facilitated cross-validation where findings converged, and a more comprehensive understanding of the findings when they were complementary. Utilization of Big CACTUS trial data in the secondary data analysis section afforded some advantages including rigorous data collection processes and a relatively large sample size for this hard to reach population. However, the limitation of using data collected for the purposes of the trial meant that important variables relating to adherence, identified in the qualitative interviews, were not measured quantitatively as the variables available were designed for the reporting of the RCT and not designed specifically for the exploration of adherence. This may explain the regression model only accounting for 29% of the variation in practice time. It is possible the model would have benefitted from the inclusion of variables such as self-efficacy (41), intrinsic motivation (42), cognitive ability (particularly executive function, which has been found to be predictive of rehabilitation participation) (43), and technology proficiency. However, finding valid quantitative measures of these variables that are accessible to PWA would be a challenge. The mean length of time between the end of the intervention period and the qualitative interviews being conducted (~8 months) might have impacted upon participants' ability to recall and reflect on the factors that influenced their adherence. However, it is worth noting that 61% of participants continued to use the computer therapy software beyond the 6-month intervention period, albeit without volunteer/assistant support (17).

Two of the factors found to be associated with adherence to aphasia computer therapy in the quantitative analysis were intervention variables: therapist time supporting the participant and length of access to computer therapy. Results from the intervention variables must be interpreted with greater caution than the demographic and clinical variables as they are not time dependent and it is possible that the amount of practice completed could have influenced the amount of support provided or length of access, rather than the other way round. There was a high variability (indicated by high standard deviations throughout), a positive skew and some outliers (particularly high adhering participants) in the dependent variable. Regression can be particularly sensitive to outliers, but as there was no evidence that the data were inaccurate outliers were not removed. The square root sensitivity analysis reduced the variability, the skew and the impact of outliers, thus improving the normality of the data (44). The process of bivariate testing used to select variables for inclusion in the regression model is criticized by some statisticians for increasing the likelihood of an “overfitted” model with an increased risk of a type I error (45). However, due to the lack of prior research around predictors of adherence to speech and language therapy interventions for aphasia there was no prior evidence or theory upon which the decision of which factors to include could be made. Conclusions must be interpreted in light of the fact that this was exploratory research.

Considerably more male PWA (83%) were recruited to the qualitative interviews. The Big CACTUS trial had a slight male gender bias 60% (17); contrary to a recent review which found that aphasia rates are higher in women (46). The increased gender difference in this sample compared to the wider Big CACTUS sample might have been due to self-selection or it might have been the result of using a maximum variation sampling strategy as it might have been that women were more often moderate adherers.

## Conclusion

Amounts of self-managed practice of aphasia therapy exercises on a computer are hugely variable from person to person. This exploratory mixed methods study highlighted a number of factors found to be associated with adherence to self-managed aphasia therapy on a computer. Clinicians delivering this asynchronous tele-rehabilitation intervention should consider the factors highlighted in this study relating to capability, opportunity and motivation when deciding which patients may be most likely to engage with this mode of treatment, as well as how they can be supported to optimize the amount of practice they engage in.

## Data Availability Statement

The raw data supporting the conclusions of this article will be made available by the authors, without undue reservation.

## Ethics Statement

Ethical approval for the secondary analysis was obtained from Leeds West Research Ethics Committee (REC; 13/YH/0377) and the Scottish A REC (14/SS/0023). Separate approval for the qualitative interviews was obtained from the School of Health and Related Research REC at the University of Sheffield (008063).

## Author Contributions

MH, RP, and CC contributed to conception and design of the study. MH performed the statistical analysis, collected the qualitative data, coded the qualitative data, and wrote the first draft of the manuscript. All authors contributed to the interpretation of the qualitative data and contributed to the article and approved the submitted version.

## Conflict of Interest

MH reports a Fellowship from the Stroke Association which funded the submitted study. RP and CC report Health Technology Assessment funding from the National Institute for Health Research (NIHR) for the Big CACTUS trial, which the submitted study was carried out alongside. RP also reports a NIHR Senior Clinical Academic Lectureship.
